# Exenatide in obesity with accelerated gastric emptying: a randomized, pharmacodynamics study

**DOI:** 10.14814/phy2.12610

**Published:** 2015-11-05

**Authors:** Andres Acosta, Michael Camilleri, Duane Burton, Jessica O’Neill, Deborah Eckert, Paula Carlson, Alan R Zinsmeister

**Affiliations:** 1 Division of Gastroenterology and Hepatology, Department of Medicine, Clinical Enteric Neuroscience Translational and Epidemiological Research (C.E.N.T.E.R.), Mayo ClinicRochester, Minnesota; 2 Division of Biomedical Statistics and Informatics, Department of Health Sciences Research, Mayo ClinicRochester, Minnesota

**Keywords:** Glucagon-like peptide 1, pharmacogenomics, satiation

## Abstract

Obesity is associated with differences in satiety, gastric emptying (GE), gastric volume, and psychological traits. Exenatide, a short-acting glucagon-like peptide 1 (GLP-1) receptor agonist, is associated with variable weight loss. We compared the effects of exenatide, 5 *μ*g, and placebo SQ, twice daily for 30 days on GE of solids and liquids (scintigraphy), satiety (ad libitum buffet meal), satiation (nutrient drink test, maximum tolerated volume [MTV]), and weight loss in 20 participants with documented accelerated GE of solids (T_1/2_ < 90 min). Exenatide delayed GE of solids (T_1/2_ [Δ] 86 min relative to placebo, *P* < 0.001) and reduced calorie intake at buffet meal ([Δ] 129 kcal compared to placebo). Median weight loss was −0.95 kg (IQR −0.7 to −2.1) for exenatide and −0.55 kg (0.3 to −2.1) for placebo (*P* = 0.23); 80% of exenatide group had documented reduction in weight. In the exenatide treatment group, there was an inverse correlation between gastric emptying T_1/2_ and MTV (*R* = −0.548, *P* = 0.089). The univariate association of weight change with posttreatment MTV was borderline (*R*s = 0.43, *P* = 0.06); in the multiple regression model, posttreatment MTV was associated with weight change (*P* = 0.047). The effect of the short-acting GLP-1 receptor agonist, exenatide, on GE is associated with the change in food intake, and the latter impacts weight loss in response to exenatide treatment.

## Introduction

Obesity prevalence continues to increase worldwide (Ng et al. 2014) and, in the United States, 69% of adults are overweight or obese (Flegal et al. 2012). There is still a lack of effective, long-term, noninvasive treatments for obesity. The current one treatment fits all approach to obesity is associated with highly variable efficacy and outcomes (Acosta et al. 2014a).

The incretin, glucagon-like peptide 1 (GLP-1), enhances glycemic control, but it impairs gastric emptying and increases satiation in health and in diabetes (Delgado-Aros et al. 2002; Peters 2010; van Can et al. 2014). GLP-1 and GLP-1 agonists reduce fasting and postprandial glucose levels. Exenatide (exendin-4) is a 39-amino acid peptide that is produced in the salivary gland of the Gila monster lizard. It has 53% homology with GLP-1, but its half-life is prolonged by avoiding the rapid breakdown by dipeptidyl peptidase 4 (DPP-IV). Exenatide, in both daily and weekly formulations, has been approved by the FDA for treatment of patients with type 2 diabetes mellitus who are inadequately controlled by treatment with metformin or sulfonylureas. GLP-1 receptor agonists also retard gastric emptying and decrease food intake by 19% (Verdich et al. 2001; Bray and Ryan 2014). Effects of short-acting exenatide on gastric emptying are temporally associated with reduced postprandial glycemia in patients with type 2 diabetes mellitus (Linnebjerg et al. 2008). Linnebjerg et al. noted a significant inverse correlation between gastric emptying T_1/2_ of solids and the 0- to 3-h postprandial blood glucose AUC; the proportion of the variance in blood glucose attributable to the delay in gastric emptying was about 25% (*r* = −0.49) (Linnebjerg et al. 2008).

Studies with GLP-1 agonists have shown an unexplained high variability of weight loss in response to treatment. Thus, treatment with exenatide, 5 *μ*g SQ twice daily, resulted in weight loss that varied from 2.0 ± 2.8 to 5.1 ± 0.5 kg in 12–24 weeks’ studies (Moreno et al. 2012). In a crossover, 16-week, per treatment arm, placebo-controlled study of exenatide for weight loss, only 30% of patients lost more than 5% of body weight (responders), whereas 70% lost less than 5% of body weight (nonresponders) (Dushay et al. 2011). Visual analog scale scores showed that nausea increased during exenatide treatment compared to placebo, with the highest scores at week 4, but they were not significantly different from placebo at week 16.

Indeed, using a relatively insensitive measurement of gastric emptying of liquids based on plasma acetaminophen appearance, it has been shown that exenatide retards gastric emptying and contributes to reduction in postprandial glycemia (Cervera et al. 2008). Similarly, the GLP-1 agonist, liraglutide, retards gastric emptying of liquids during the first postprandial hour (van Can et al. 2014).

We recently observed, in a study of 509 normal weight, overweight, or individuals with obesity, that obesity is associated with different gastrointestinal traits: decreased satiation, accelerated gastric emptying, larger fasting gastric volume, and decreased peak postprandial plasma peptide YY level (Acosta et al. 2015b). In a principal components analysis, we identified latent dimensions accounting together for 81% of overweight and obesity variance, including four main latent dimensions: satiety (21%), gastric capacity (14%), psychological (13%), and gastric motor-sensory functions (11%).

Our overall hypothesis was that biomarkers such as accelerated gastric emptying of solids in obesity may influence the response to GLP-1 agonists such as exenatide or liraglutide, which are being tested as weight loss remedies in obesity (Bray and Ryan 2014). The ultimate goal is to identify endophenotypes or quantitative traits, as well as obesity-related genotypes to enhance the efficacy of pharmacotherapies for weight loss.

Our specific hypothesis in this study was that exenatide retards gastric emptying in obese patients with baseline accelerated gastric emptying. Thus, we conducted a randomized, controlled trial to compare the effects of exenatide and placebo in adult patients with obesity and previously documented accelerated gastric emptying; we also appraised the effects of exenatide on quantitative traits that were associated with weight loss.

## Materials and Methods

### Study design

We performed a double-blinded, placebo-controlled, randomized clinical trial of twice daily, subcutaneous (SQ) exenatide, 5 *μ*g, or placebo administered for 30 days in patients with obesity in whom there was documented accelerated gastric emptying of solids. Thus, we invited for this study 58 individuals with obesity (BMI 30–40 kg/m^2^) and known accelerated gastric emptying (GE) of solids (GE T_1/2_ < 79 min or GE 1 h >35%) who had participated in recent studies (Acosta et al. 2014b, 2015a,b). We selected for randomization in this study the first 20 subjects who agreed to participate. Ten participants were randomized to each treatment arm according to a computer-generated randomization schedule by the study statistician’s office and submitted to Mayo Clinic Research Pharmacy. Allocation was concealed. The study blind was retained until all the data (gastric emptying, satiation and satiety, weight and waist circumference) had been recorded or analyzed and locked in a “blinded database” by the statistician.

After written informed consent, participants completed questionnaires and underwent a physical examination; women of childbearing potential were required to have a negative urine pregnancy test within 48 h prior to tests involving radioactivity. Participants were instructed to continue on the same diet and exercise routine during the entire therapeutic trial. Study participants presented to the testing facility (Mayo Clinic Clinical Research Unit, Charlton Building, 7th floor) after overnight fast for all studies. We performed a baseline satiation on Day 0 prior to initiation of medications or placebo; the gastric emptying test by scintigraphy, satiety, and nutrient drink satiation tests were performed in the last 5 days of the 30 days of medication administration.

### Participants

The main inclusion criteria were adults 18 years or older, obesity class I and II (BMI 30–40 kg/m^2^), and accelerated GE T_1/2_ < 79 min or GE 1 h >35%. The latter criteria for accelerated gastric emptying were based on published data in 319 healthy participants (Camilleri et al. 2012). We excluded patients with chronic conditions such as diabetes mellitus or gastroparesis, patients with unstable weight for the last 3 months, and patients taking weight loss medications or medications that may alter gastric emptying.

### Study medication

Exenatide (Byetta®, Amylin Pharmaceutical, San Diego, CA) was purchased by Mayo Clinic Research Pharmacy. Placebo for subcutaneous injection was prepared by the Mayo Clinic Research Pharmacy and consisted of normal saline (0.9%). The dose of 5 *μ*g b.i.d. was selected based on the recommended starting dose in the FDA-approved package insert (http://www.accessdata.fda.gov/drugsatfda_docs/label/2009/021773s9s11s18s22s25lbl.pdf).

### Gastric emptying of solids

One mCi ^99m^Tc-sulfur colloid was added to two raw eggs during the scrambling, cooking process. The eggs were served on one slice of bread with 240 mL of skim milk (total calories: 296 kcal, 32% protein, 35% fat, 33% carbohydrate). Anterior and posterior gamma camera images were obtained immediately after radiolabeled meal ingestion, every 15 min for the first 2 h, then every 30 min for the next 2 h (total 4 h after the radiolabeled meal) (Vazquez-Roque et al. 2006). Data were analyzed as in previous studies (Cremonini et al. 2002). Geometric mean of decay-corrected counts in anterior and posterior gastric regions of interest was used to estimate the proportion of ^99m^Tc emptied at each time point (gastric emptying). Normal data and performance characteristics based on studies in 319 healthy controls have been previously reported (Camilleri et al. 2012).

### Satiation test

We used a standardized and validated nutrient drink test (Chial et al. 2002) to measure satiation and postprandial symptoms in relation to drinking a liquid nutrient at constant rate (30 mL/min Ensure®: 1 kcal/mL, 11% fat, 73% carbohydrate, and 16% protein). Subjects scored the level of fullness or satiation using a scale that combines verbal descriptors and numbers (0 = no symptoms; 3 = usual fullness; 5 = maximum tolerated volume or unbearable fullness/satiation). Nutrient intake was stopped when subjects reached the score of 5. Postprandial symptoms of fullness, nausea, bloating, and pain were measured 30 min after the meal using 100 mm horizontal visual analog scales (VAS), anchored with “none” and “worst ever” at the left and right ends of the lines for each symptom.

### Standard buffet meal to assess satiety

The standard buffet meal was served 5 h after ingestion of the standard egg meal ingested to measure gastric emptying. This approach has been successfully used in studies to measure caloric intake (Acosta et al. 2015b). The items of the standard buffet were purchased and calories ingested were calculated. The ad libitum meal items were (per serving): Stouffers® lasagna (450 calories, 22% protein calories, 46% carbohydrate calories, 32% fat calories), Stouffers® vegetable lasagna (420 calories, 16% protein calories, 37% carbohydrate calories, 47% fat calories), cartons of Kraft® pudding (120 calories, 2% protein calories, 76% carbohydrate calories, 22% fat calories), and skim milk (90 calories, 36% protein calories, 64% carbohydrate calories, 0% fat calories). Personnel from the study team weighed the food servings postmeal and reported the amount of food left from single portions partially consumed.

### Safety assessments

Safety was determined by evaluating adverse events, vital signs, and physical examinations at baseline and standard times postdosing.

### Study endpoints, statistical methods, and data analysis

Data are generally expressed as median (IQR) unless otherwise stated.

#### Endpoints

The primary endpoint for analysis was gastric emptying T_1/2_ of solids during treatment.

Secondary endpoints were proportion of gastric content emptied at 1 h; weight loss at Day 30 (change from baseline); satiation expressed as volume to fullness and maximum volume of Ensure® ingested (nutrient drink test); caloric intake from a standard buffet meal (satiety test); and aggregate symptom score 30 min after ingestion of maximal tolerated volume.

#### Statistical analysis

The effects of exenatide on transit parameters, satiation, symptom scores, and satiety were assessed using analysis of covariance (ANCOVA) models, with the measured endpoints as the response (dependent variable), adjusting for the relevant covariates (e.g., gender, BMI). In addition, the nutrient drink test (volume to fullness and maximal tolerated volume [MTV]) measured at baseline (prior to treatment) was used as covariate in the assessment of effects of treatment on satiation (the MTV and the aggregate symptom scores 30 min after meal ingestion).

Although not the primary analysis of the study, we compared data (e.g., weight, waist circumference, satiation parameters, gastric emptying T_1/2_) between on-treatment and baseline or, in the case of gastric emptying, prior study that was used for eligibility to participate in the current treatment trial. These analyses followed the intent-to-treat (ITT) paradigm, including all subjects randomized.

#### Sample size assessment

Table1 summarizes data for primary response measures and uses the relative variation (CV%) to estimate the effect size detectable with 80% power based on a two-sample *t*-test at a two-sided *α* level of 0.05. The effect size is the difference in group means as a percentage of the overall mean for each response and assumes 10 subjects per group; these effect sizes (difference in gastric emptying T_1/2_ solids at 40 min based on normal values of T_1/2_ 121.7 ± 29.8 min [coefficient of variation, COV, 23.8%] or 12.5% difference in the percent emptied from the stomach at 1 h, based on normal values of 18.1 ± 9.5% [COV 52.7%]) are clinically significant, as they reflect a change in gastric emptying from the mean observed in 319 healthy controls previously reported using the same method from our laboratory (Camilleri et al. 2012). These estimates were based on calculations performed using SigmaPlot® Software.

**Table 1:** Sample size and power analysis, assuming *n* = 10 per treatment group

Response type	Mean	SD	CV%	Effect size [Δ] with 80% power
Solid gastric emptying T_1/2_ (min)	121.7	29.8	23.8	40 min
Solid gastric emptying at 1 h (%)	18.1	9.5	52.7	12.5%

#### Evaluation of factors associated with weight loss

The associations of weight change (post minus pre) with gastric emptying (GE at 60 min, GE T-Lag, and GE T_½_) and maximum tolerated volume (baseline, posttreatment, and delta [post minus pre]) were assessed univariately using Spearman’s correlation coefficients. In addition, two multiple regression models were examined: (1) weight change versus predictors (GE T–Lag and GE T_½_); and (2) weight change versus predictors (post MTV and delta MTV [post minus pre]).

## Results

### Participants, demographics, and baseline parameters

**Figure 1:**
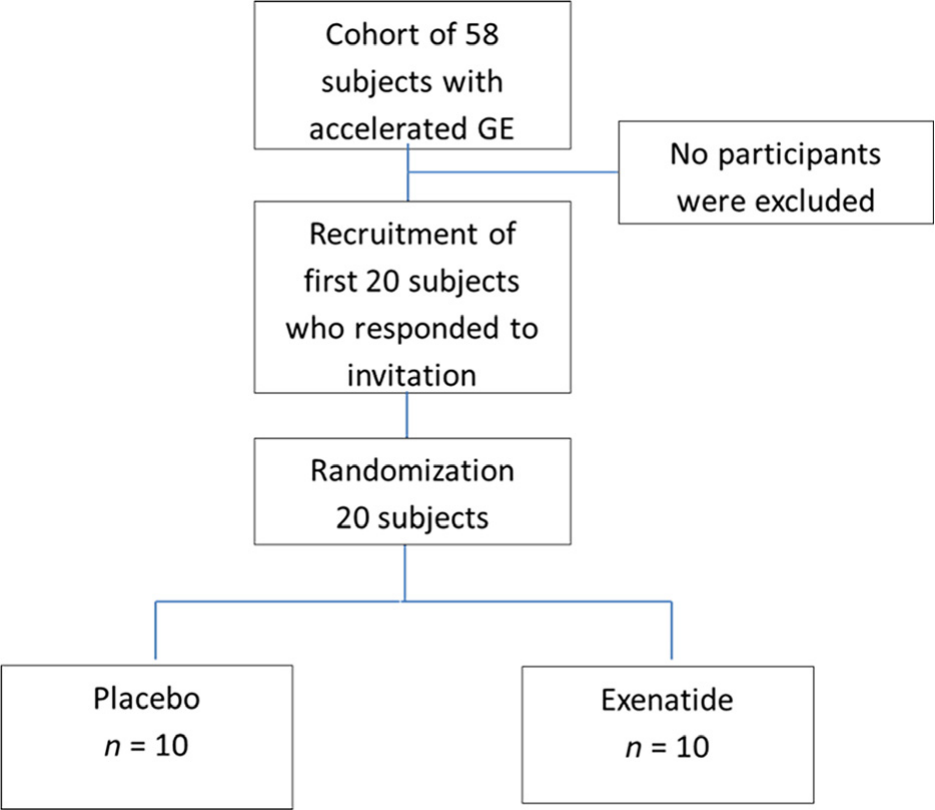
Consort diagram.

Figure1 shows the CONSORT flowchart for the study. Fifty-eight participants were invited to participate, and the first 20 participants to respond to the invitation were screened and enrolled in the study. The characteristics of the participants were 65% female, predominantly Caucasian (90%), mean (±SEM) age 39.3 ± 3.7 years, and BMI 33.9 ± 1 kg/m^2^ (Table2). Demographics, baseline nutrient drink test, and results of prior gastric emptying test used to qualify participants for the study were similar in both treatment groups (Table2).

**Table 2:** Demographics and baseline characteristics

	Placebo	Exenatide
N	10	10
Age (y)	42.5 (25.3–49.5)	34 (33–46.8)
Gender, female (%)	70	60
Ethnicity, Caucasian/Hispanic (%)	90/10	90/10
Body weight (kg)	103.4 (87.6–111.9)	98.1 (89.4–109.7)
BMI (kg/m^2^)	34.2 (31.7–38.5)	33.5 (32.9–36.7)
Waist circumference (cm)	108.5 (105–118)	107.7 (100–121)
Solid GE: proportion emptied at 1 h	43.5 (36–51.9)	41.8 (35.1–45.1)
Solid GE T_½_ min	77.6 (56.4–94.2)	79.4 (70.1–98.1)
Volume to fullness, mL (mL)	630 (412–975)	690 (413–975)
Maximum tolerated volume (mL)	1185 (941–1422)	1304 (1035–1807)
Aggregate satiation symptoms (VAS, mm)	160 (118–247)	199.5 (121–235)
Nausea (VAS, mm)	20 (4.8–58.8)	28.5 (10.3–58)
Fullness (VAS, mm)	66.5 (47–79)	73 (60.5–82.8)
Bloating (VAS, mm)	64 (26.5–78.3)	62 (40.3–70)
Pain (VAS, mm)	28 (7.8–70)	22 (6–30.3)

Data are median (IQR). Data on gastric emptying (GE) are from same participants in prior studies; symptoms appraised using 100-mm visual analog scales (VAS).

### Effect of exenatide on gastric emptying

**Figure 2:**
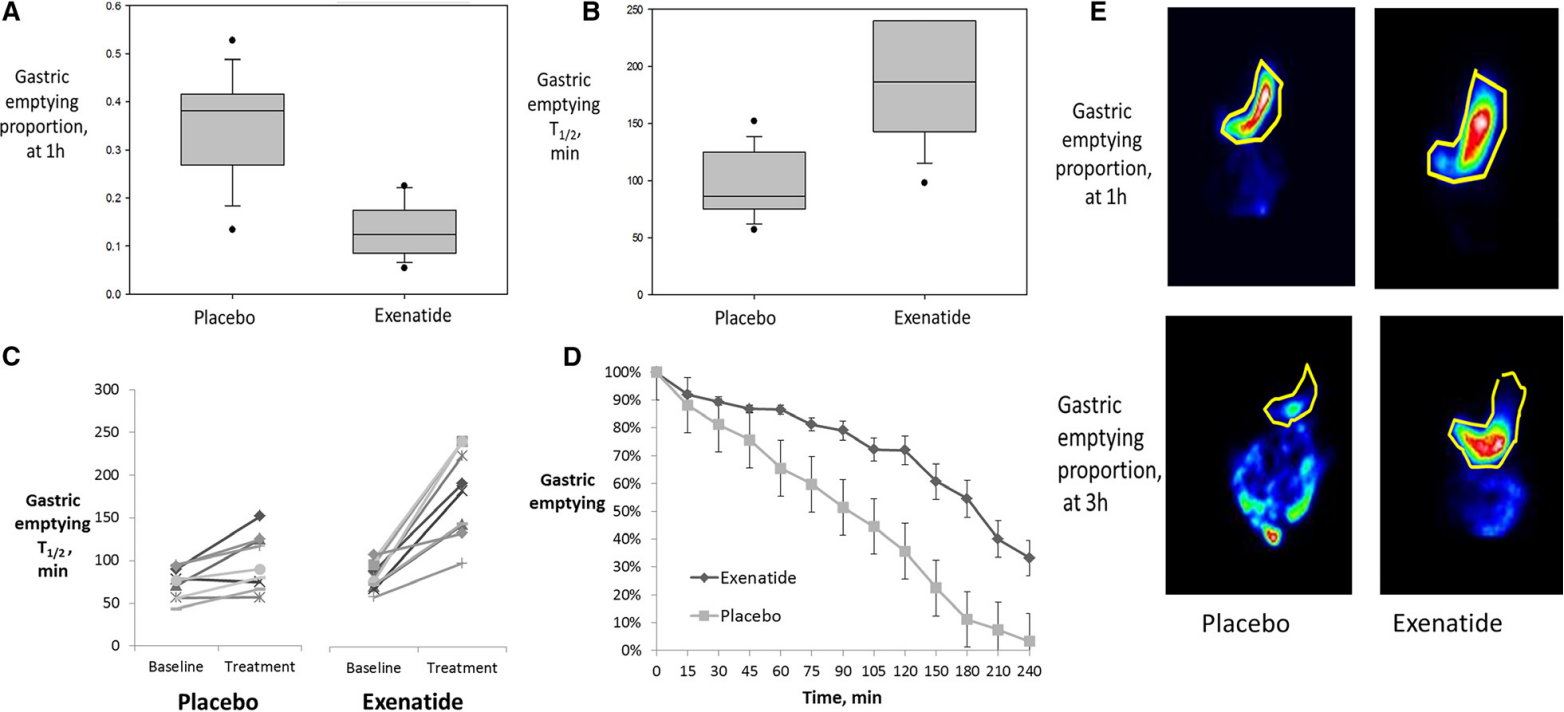
Effect of exenatide on gastric emptying in obese patients with previously documented accelerated gastric emptying (*P* < 0.001 for both [A] gastric emptying proportion at 1 h and [B] gastric emptying T_1/2_). Data show median, IQR, and range. (C) Each individual’s gastric emptying measurements at prior study on no treatment (“baseline”) and on treatment in the current study are plotted. (D) The rate of gastric emptying per group. (E) An illustrative scintigraphic image of gastric emptying at 3 h in placebo versus exenatide, with stomach area demarcated in yellow.

Exenatide significantly delayed the gastric emptying of solids when compared to placebo (*P* < 0.001 for GE at 1 h and GE T_1/2_) (Fig.2A, B). The proportion emptied from the stomach at 1 h was 12.4% (8–18.5) in the exenatide group versus 38.2% (26.6–42.1) in the placebo group, and the GE T_1/2_ was 187 min (141–240) in the exenatide group compared to 86 min (73–125) in the placebo group (Fig.2C, D and Table3). There was no significant difference in the placebo group compared to baseline. Data from each participant in the exenatide and placebo groups are shown in (Fig.2C); two of the participants receiving exenatide appeared not to respond as well to the exenatide treatment. Figure2E is a representative scintigraphy image comparing both groups at 1 h and T_1/2_.

**Table 3:** Effects of exenatide on obesity-related gastrointestinal traits

	Placebo	Exenatide	*P*
Posttreatment body weight (kg)	103 (87–108)	96 (87–108)	ns
Change in body weight (kg)	−0.55 (0.3 to −2.1)	−0.95 (−0.7 to −2.1)	ns
Posttreatment waist circumference (cm)	107.4 (104–119)	104.7 (97–121)	ns
Change in waist circumference (cm)	−0.35 (1.4 to −2.1)	−3.65 (0.55 to −7)	0.06
Solid GE proportion emptied at 1 h (%)	38.2 (26.6–42.1)	12.4 (8–18.5)	<0.001
Solid GE T_1/2_ (min)	86 (73–125)	187 (141–240)	<0.001
Volume to fullness (mL)	675 (413–825)	705 (510–795)	ns
Maximum tolerated volume (mL)	1052 (859.1–1422)	1244 (918–1452)	ns
Aggregate satiation symptoms (VAS, mm)	172 (110–237)	145 (133.5–204)	ns
Nausea (VAS, mm)	13 (3–73.5)	43 (15.3–62.5)	ns
Fullness (VAS, mm)	61 (47.5–74.3)	63 (49.5–84.8)	ns
Bloating (VAS, mm)	62.5 (38–75)	39 (23–67.8)	ns
Pain (VAS, mm)	24.5 (3–46.8)	21 (8–34)	ns
Buffet meal intake (kcal)	1110 (680.3–1658)	977 (713–1336)	ns
Protein intake (%)	21.6 (18.1–22.2)	23 (21.9–24)	ns
Fat intake (%)	20.5 (18.3–23.1)	20.5 (18.9–22.9)	ns
Carbohydrate intake (%)	58.6 (55–59.7)	55.6 (54.5–58.8)	ns

Data are median (IQR). ns, not significant; GE, gastric emptying; VAS, visual analog scale.

### Effect of exenatide on body weight

**Figure 3:**
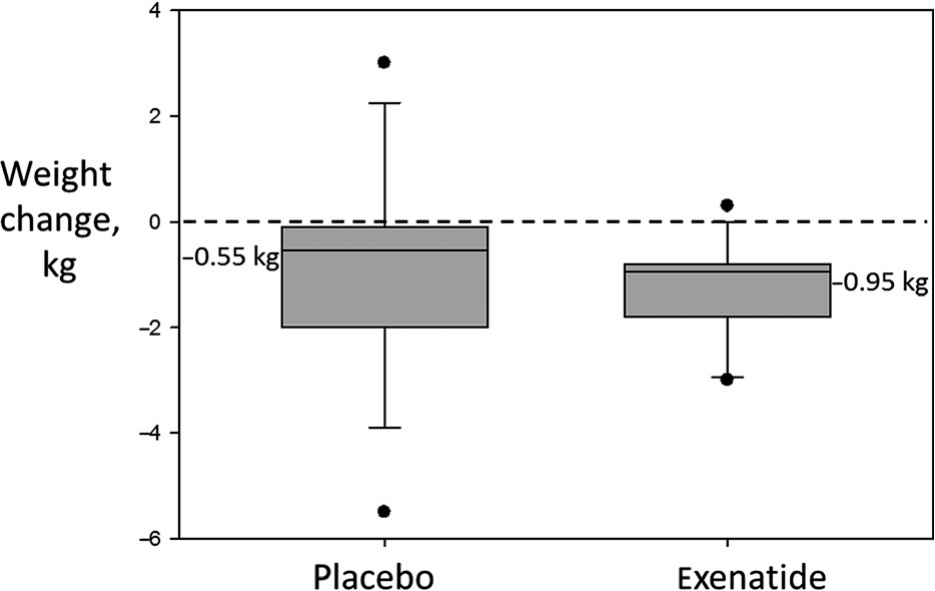
Effect of exenatide on body weight. Data shown are medians (IQR).

Patients treated with exenatide lost 0.95 kg (−0.7 to −2.1) compared to 0.55 kg (0.3 to −2.1) in the placebo group (*P* = 0.23 between groups posttreatment, Fig.3). The weight change in the exenatide-treated group was significantly different when compared to baseline (*P* = 0.004). There was no significant difference in the placebo group compared to baseline.

### Effect of exenatide on waist circumference

In the exenatide group, 80% of patients lost more than 0.8 kg in 30 days compared to 50% in the placebo group. Patients treated with exenatide had a reduction in waist circumference of −3.65 cm (0.55 to −7.0) compared to −0.35 cm (1.4 to −2.1) in the placebo group (*P* = 0.06 between groups). The waist circumference in the exenatide-treated group was significantly different when compared to baseline (*P* = 0.04). There was no significant difference in the placebo group compared to baseline (Table3).

### Effects of exenatide on satiety and satiation

During the satiety buffet meal, patients treated with exenatide consumed 123 kcal less than patients on placebo; this numerical difference was not statistically significant.

During the satiation test, volume to fullness and maximum tolerated volume (MTV) were not significantly different on exenatide compared to placebo; however, patients treated with exenatide consumed 119 mL (0–356) less than at baseline (*P* = 0.01), while there was no significant difference in patients on placebo (difference 0 mL [30–289], *P* = 0.13). There were no differences in postprandial symptoms in either group compared to baseline.

### Factors that predict weight loss in patients with accelerated gastric emptying

**Figure 4:**
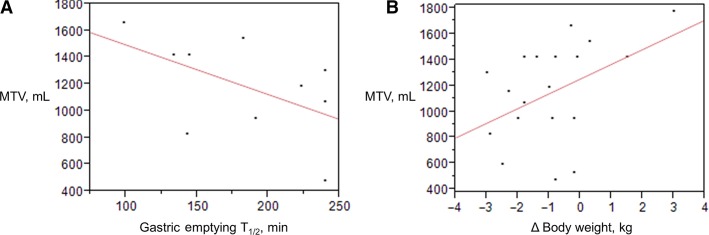
(A) Relationship of gastric emptying and maximum tolerated volume (MTV) in patients treated with exenatide (−*r* = −0.548; *P* = 0.089). (B) In a multiple regression model, including gastric emptying and satiation parameters, posttreatment MTV was modestly associated with weight change (*P* = 0.047).

In the exenatide treatment group, there was an inverse correlation between gastric emptying T_1/2_ and MTV (*R* = −0.548, *P* = 0.089) (Fig.4A). The univariate association of weight change with posttreatment MTV was borderline (Spearman = 0.43, *P* = 0.06), while in the multiple regression model, posttreatment MTV was modestly associated with weight change (*P* = 0.047) (Fig.4B).

### Adverse events

Overall, exenatide was well tolerated and there were no deaths or adverse events. All the participants completed the study.

## Discussion

In this randomized pharmacodynamics trial of 30 days’ treatment with the GLP-1 agonist, exenatide, compared to placebo, significantly delayed the gastric emptying of solids, and produced a nonsignificant reduction in body weight and decreased caloric intake in a buffet meal test when compared to placebo. There was an inverse correlation between the gastric emptying T_1/2_ and the MTV during a satiation test. In a multiple regression model, posttreatment MTV was associated with weight change. These data are consistent with the concept that an effect of exenatide on gastric emptying may contribute to the weight loss observed in larger, longer duration, randomized, placebo-controlled trials (Moreno et al. 2012). These results are also consistent with effects of exenatide on gastric emptying which are temporally associated with reduced postprandial glycemia in patients with type 2 diabetes mellitus (Linnebjerg et al. 2008). However, this is a first demonstration of the persistence of marked slowing of gastric emptying at 30 days’ treatment with exenatide, demonstrated by gold standard methods, that is, radioscintigraphic emptying of a solid meal, in contrast to a prior study based on a paracetamol test (DeFronzo et al. 2008) that measures gastric emptying of liquids.

The pharmacological treatment of obesity is associated with high variability in weight loss and occurrence of side effects. We have recently observed subgroups of patients with obesity based on different quantitative gastrointestinal and psychological traits, confirmed by an analysis of latent dimensions: satiety (21%), gastric capacity (14%), psychological trait (13%), and gastric motor-sensory functions (11%). In addition, we showed that, in a small trial of patients treated with phentermine–topiramate extended release, reduced satiety with increased calorie intake at a buffet meal predicted weight loss, suggesting that pretreatment identification of decreased satiety may be used to decrease the variability in weight loss in response to treatment with phentermine–topiramate extended release (Acosta et al. 2015b).

In the present study in patients with accelerated gastric emptying, 80% of patients treated with exenatide lost weight compared to previous randomized trials of exenatide which typically have shown approximately 30% responders (Rosenstock et al. 2010; Dushay et al. 2011; Moreno et al. 2012). Clearly, findings of this pilot study need replication in a larger, longer duration, randomized clinical trial in patients with accelerated gastric emptying. We noted that two patients on exenatide who were nonresponders had a minor change in gastric emptying (similar to that observed on placebo) and also had consumed over 2000 kcal in the satiety test, suggesting that, in addition to accelerated gastric emptying, they also had a separate physiological quantitative trait predisposing them to obesity. These data suggest the hypothesis that, in addition to acceleration of gastric emptying, the optimal weight responders to the GLP-1 agonist class of drugs among patients with obesity may be those without markedly reduced satiety (i.e., those who are unable to ingest >2000 kcal in an ad libitum meal).

There are limitations in our study, specifically the prespecified focus on individuals with accelerated gastric emptying in a prior study, which pertains to about 25% of a cohort of 274 patients with obesity. Thus, our data cannot be regarded as generalizable, but this is consistent with the overall objective to identify obese patients more likely to respond to the GLP-1 agonist class of drugs without the induction of adverse effects.

On the other hand, a strength of our study is the well-characterized subgroup in whom the observed gastric emptying of solids was at or below the 10th percentile for gastric emptying in obese individuals (average for obesity GE T_1/2_ 100 min) (Dushay et al. 2011) and at or below the 2.5th percentile of gastric emptying in healthy controls (average for healthy GE T_1/2_ 120 min) (Camilleri et al. 2012).

Exenatide treatment, in the selected population, showed a remarkable delay in gastric emptying T_1/2_ by 100 min without induction of nausea, vomiting, or worsening of postprandial symptoms (nausea, bloating, fullness, or pain) after the fully satiating liquid nutrient drink ingested to the maximum tolerated volume. These results may suggest that individuals with accelerated gastric emptying may have a deficiency in the secretion, release, or effects of hormones that normally retard gastric emptying, such as cholecystokinin, glucose-stimulated insulinotropic peptide, or GLP-1. These deficiencies can be pharmacologically reversed by the GLP-1 agonist. Prior studies suggested that GLP-1 stimulated nitrergic pathways in humans (Andrews et al. 2007); this could conceivably explain the effect of GLP-1 on enhanced gastric accommodation (Delgado-Aros et al. 2002; Schirra et al. 2002) or the inhibition of antral motor function, which would be expected to retard the trituration of the solid component of the meal, as previously demonstrated by Schirra et al. (Schirra et al. 1997). In fact, studies with the GLP-1 antagonist, exendin _9-39_ amide, showed that endogenous GLP-1 inhibited antral motility through cholinergic mechanisms (Schirra et al. 2006, 2009).

Exenatide treatment was also associated with lower food intake (median difference 133 kcal) compared to placebo. Although this difference in caloric intake was not statistically significant in this small study, it is a clinically significant difference, is greater than the effect seen in previous studies with exenatide (Rosenstock et al. 2010), and is similar to the effect of high doses of liraglutide on satiety. There was no significant difference in weight loss in the exenatide group compared to placebo group; this failure may be related to the relatively small sample size, the short duration of the study, and the impact of one placebo treated patient who lost 6.3 kg in 30 days, on the analysis of the efficacy of treatments. For the entire group of 20 participants, the mean weight reduction was 1.06 kg, and the standard deviation of weight change was 1.76 kg. Using this standard deviation and assuming a weight loss difference of 2 kg (highly optimistic efficacy) or the ∼0.5 kg (observed at 1 month of treatment in the current study), the sample size to demonstrate a significant difference in the two treatment arms would be 14 or 195 per treatment arm, respectively. We believe that the range of weight loss selected for these sample size estimates are reasonable given the published literature. Thus, for example, the estimated mean weight loss in nondiabetics treated with exenatide for 1 month was ∼1.5 kg (Dushay et al. 2011); and a systematic review and meta-analysis of 25 clinical trials of at least 20 weeks’ duration with GLP1R agonists (involving exenatide 5 *μ*g twice daily, 2 mg exenatide once weekly, or 1.2 mg liraglutide once daily) showed a weighted mean difference compared to control groups of −2.9 kg greater with the GLP1R agonists (−3.2 kg in nondiabetics and −2.8 kg in diabetics) (Vilsbøll et al. 2012). The range of maximum treatment efficacy observed in our current study (3.0 kg in the exenatide group and, paradoxically, 6.3 kg in the placebo group) also emphasizes the importance of performing randomized, placebo-controlled trials in addition to sample size considerations.

Finally, the effects with a short-acting GLP-1 receptor agonist cannot be extrapolated to long-acting GLP-1 agonists, which have a stronger effect on fasting glucose levels mediated predominantly through their insulinotropic and glucagonostatic actions (Meier 2012). In fact, the impact of the reduction in 1-h gastric emptying of liquids by 23% with liraglutide, 3.0 mg, compared to placebo (van Can et al. 2014) is of unclear clinical significance and does not necessarily imply that the long-acting GLP-1 agonist, liraglutide, would retard the gastric emptying of solids. Importantly, mechanistic studies in a rat model (Jelsing et al. 2012) showed that the gastric inhibition by GLP-1 receptor agonists is subject to desensitization/tachyphylaxis, but this effect is dependent on exposure over the full 24-h period to the GLP-1 receptor agonist, and this may not be achieved by exenatide administered twice daily as in the current study. Thus, gastric emptying of a radiolabeled mashed potato meal was markedly slowed by acute GLP-1 administration, though the magnitude of the slowing was attenuated with prolonged 24-h infusions, but was maintained with intermittent infusions (Umapathysivam et al. 2014). Overall, the observation by the Adelaide group (Umapathysivam et al. 2014) that twice daily exenatide administered for 1 day delays gastric emptying of a mashed potato meal is consistent with the results of our study of the emptying of a 320 kcal, 30% fat, radiolabeled, solid plus liquid meal after 30 days of exenatide administration twice daily. While the mashed potato meal probably does not require trituration and may, therefore, not be significantly altered as a result of inhibition of antral motility by the GLP-1 receptor agonist (Schirra et al. 1997, 2002, 2006, 2009; Rosenstock et al. 2010), our study of gastric emptying of solids demonstrated the marked inhibition of early (gastric emptying at 1 h) and overall gastric emptying by exenatide. Whereas, we did not study antral motility by simultaneous manometry in the current study, these observations on gastric emptying suggest that exenatide may inhibit antral motor function during the trituration period (lag phase), as previously documented by Schirra et al. with native GLP-1 (Schirra et al. 1997).

Therefore, these data would suggest that there may not be a retardation of gastric emptying of solids with chronic administration of a long-acting GLP-1 receptor agonist such as liraglutide. In addition, rat studies convincingly demonstrate that body weight-lowering effects of GLP-1 receptor stimulation are not subject to desensitization, suggesting that regulation of appetite signals in the brain, and not effects on gastric emptying, constitutes the main mechanism for liraglutide-induced weight loss (Jelsing et al. 2012). The inference is that there is a good rationale for selection of patients with accelerated gastric emptying for treatment with the short-acting GLP-1 receptor agonists such as exenatide or lixisenatide which retard gastric emptying (Meier et al. 2015), but not with the longer lasting GLP-1 receptor agonist, liraglutide. Formal studies comparing effects of these different GLP-1 receptor agonists in patients with accelerated or normal gastric emptying are eagerly awaited.

## Conclusions

The effects of the short-acting GLP-1 receptor agonist, exenatide, on gastric emptying are significant in obese patients with accelerated gastric emptying of solids; these effects are inversely correlated with calorie intake on satiation test, and the latter is correlated with effects on reduction in body weight. Identifying accelerated gastric emptying in patients with obesity may be an approach to enhance pharmacological response to the GLP-1 agonist, while minimizing side effects. This pilot study provides the preliminary data to plan a larger, longer duration, randomized clinical trial to replicate these observations.
